# Prevalence of Horizontal Violence Among Emergency Attending Physicians, Residents, and Physician Assistants

**DOI:** 10.5811/westjem.2016.10.31385

**Published:** 2017-01-19

**Authors:** Nico B. Volz, Ryan Fringer, Bradford Walters, Terry Kowalenko

**Affiliations:** * Oakland University William Beaumont School of Medicine, Department of Emergency Medicine, Auburn Hills, Michigan; † Beaumont Health, Department of Emergency Medicine, Royal Oak, Michigan

## Abstract

**Introduction:**

Horizontal violence (HV) is malicious behavior perpetrated by healthcare workers against each other. These include bullying, verbal or physical threats, purposeful disruptive behavior, and other malicious behaviors. This pilot study investigates the prevalence of HV among emergency department (ED) attending physicians, residents, and mid-level providers (MLPs).

**Methods:**

We sent an electronic survey to emergency medicine attending physicians (n=67), residents (n=25), and MLPs (n=24) in three unique EDs within a single multi-hospital medical system. The survey consisted of 18 questions that asked participants to indicate with what frequency (never, once, a few times, monthly, weekly, or daily) they have witnessed or experienced a particular behavior in the previous 12 months. Seven additional questions aimed to elicit the impact of HV on the participant, the work environment, or the patient care.

**Results:**

Of the 122 survey invitations 91 were completed, yielding a response rate of 74.6%. Of the respondents 64.8% were male and 35.2% were female. Attending physicians represented 41.8%, residents 37.4%, and MLPs 19.8% of respondents. Prevalence of reported behaviors ranged from 1.1% (Q18: physical assault) to 34.1% (Q4: been shouted at). Fourteen of these behaviors were most prevalent in the attending cohort, six were most prevalent in the MLP cohort, and three of the behaviors were most prevalent in the resident cohort.

**Conclusion:**

The HV behaviors investigated in this pilot study were similar to data previously published in nursing cohorts. Furthermore, nearly a quarter of participants (22.2%) indicated that HV has affected care for their patients, suggesting further studies are warranted to assess prevalence and the impact HV has on staff and patients.

## INTRODUCTION

Disruptive behaviors, such as bullying, verbal or physical threats, emotional abuse, and other purposeful malicious acts initiated by one co-worker and aimed toward another, are often termed horizontal violence (HV) or lateral violence. Prevalence research in healthcare has thus far been studied almost exclusively in the context of nursing.^1^–^5^ These behaviors negatively impact patient care and safety, increase hospital costs, decrease the morale of the healthcare team, and negatively impact the health and wellbeing of those involved.^1^,^6^,^7^ Based on a study performed by the Institute for Safe Medication Practices (ISMP), which included 2,095 healthcare providers, almost half (49%) stated that they altered how they clarify medication orders based on previously encountered intimidating behaviors.^8^ Another study found that 17% of 1,441 respondents were aware of a specific adverse event, defined as “an injury resulting from a medical intervention not due to the underlying condition of the patient,” which occurred due to disruptive behavior.^9^ Unlike violence originating from patients toward staff, as previously studied, HV is more subtle, frequently non-physical, and ambiguous.^10^ In 2004 Dr. Griffin described the 10 most common forms of lateral violence as “Nonverbal innuendo (raising eyebrows, face-making),” “Verbal affront (covert or overt, snide remarks, lack of openness, abrupt responses),” “Undermining activities (turning away, not available),” “Withholding information (practice or patient),” “Sabotage (deliberately setting up a negative situation),” “Infighting (bickering with peers,)” “Scapegoating (attributing all that goes wrong to one individual),” “Backstabbing (complaining to others about an individual and not speaking directly to that individual),” “Failure to respect privacy,” and “Broken confidences.” 11

For consistency the questions chosen in this survey are similar to questions that have previously been used in HV research among nurses and aimed to address some of the 10 most common behaviors identified by Griffin. Prevalence data varies greatly among studies due to inconsistent measurement techniques and subjective reporting. In the United States estimates suggest that prevalence is between 5%–38%.^2^ In response, the Joint Commission has produced a sentinel event alert (SEA) in an effort to improve patient and staff safety, wellbeing, and working conditions.^1^ In this study we asked participants to respond whether they had witnessed or experienced HV behaviors originating from a co-worker toward themselves or toward another co-worker.

The goal of this pilot study was to assess whether HV extends beyond the nursing context and whether future studies are warranted to further identify disruptive behaviors and eventually aim to improve the work environment and patient care. To the best of our knowledge this is the first study looking at the prevalence of HV among emergency medicine (EM) attending physicians, residents, and MLPs.

## METHODS

### Study Design

This study implemented a descriptive cross-sectional design to ascertain the prevalence of HV in a population of attending physicians, residents, and physician assistants. All participants were current employees of a single practice plan who staffed three hospitals that are part of a large multicenter system in suburban Detroit, MI. We used an anonymous electronic survey using SurveyMonkey (www.surveymonkey.com), and distributed a link to the survey via e-mail. All data were collected electronically and anonymously between the dates of November 24, 2014, and January 1, 2015. We sent three follow-up e-mails via the electronic survey provider to participants who had not responded. To increase the response rate, participants who completed the survey were provided with a link to a second and separate survey to collect e-mail addresses that were then entered into a lottery system for a chance to win a $100 VISA gift card. Survey responses and e-mail addresses of participants were not linked, thus maintaining complete anonymity. One e-mail address was chosen at random by a randomizing algorithm provided by randomresult.com as the winner of the gift card. This study was reviewed and approved by the local health system internal review board committee.

### Selection and Participant Demographics

Any participant who was a current employee (physician, resident or physician assistant) in the ED of one of the three hospitals surveyed in this study and had a valid e-mail address on file was included. We contacted 122 eligible participants, of whom 56 were attending physicians, 42 residents, and 24 physician assistants. Table 1 presents the demographic data of the 91 participants of this study.

### Method of Measurement and Statistical Analysis

The survey consisted of 18 questions regarding HV (Table 2) and seven additional questions aimed to elicit its impact on the participant (Table 3). We addressed the validity of this survey by designing questions based on previous peer-reviewed studies with the same or similar endpoint. Eight of the 18 questions were based on several previously published surveys measuring prevalence of HV in the nursing context.^2^,^8^,^12^,^13^ We designed 10 of the questions used in this survey based on the Negative Acts Questionnaire - Revised (NAQ-R), a validated survey tool designed to measure the prevalence of workplace bullying.^14^ The survey was adjusted based on feedback from the statistician of the local research institute but has not been validated by other experts in the field or by a sample population. The prompt stated, “Please answer how many times in the last 12 months, on average, you have personally experienced or witnessed any of the following behaviors displayed by a coworker (ex: by a physician, nurse, PA, resident, technician, etc.).” The answer choices for the 18 behavior questions were “never,” “once,” “a few times,” “monthly,” “weekly,” or “daily.”

We considered only behavior responses of at least “a few times” or more for the purpose of prevalence analysis and discussion in this study. The data were exported via Excel and SAV formats and sent to the local research institute for statistical analysis. The primary endpoint of interest was an estimation of the prevalence of horizontal violence (Figure). We calculated analysis of prevalence in each subgroup, attending physician, resident, and MLP, along with providing 95% confidence intervals.

## RESULTS

We sent 122 survey invitations via e-mail, and 91 participants completed the electronic survey, yielding a response rate of 74.6%. Of those who responded 64.8% were male. Attending physicians represented 41.8%, residents 37.4%, mid-level providers 19.8%, and other (fellow) 1.1% of respondents.

### Prevalence of Horizontal Violence Behaviors

The prevalence of HV behavior is measured as a participant having indicated that they experienced or witnessed a particular behavior at least “a few times” or more over the 12 months prior to taking this survey. If a behavior was experienced or observed more than just one time in the preceding 12 months, the participant was asked to indicate with what frequency this behavior was experienced or observed (a few times, monthly, weekly, or daily) to further characterize its prevalence. Data for this survey ranged between very low prevalence of 1.1% (n=1) to a prevalence of 34.1% (n=31) as indicated in the Figure. We did not include prevalence data for question 3 in the discussion as it was determined not to represent horizontal violence, based on feedback as mentioned in the limitations section of this paper.

### Subjective Impact of Horizontal Violence Behavior

Participants responded to seven additional questions aimed to gauge the impact of these behaviors on their work and personal wellbeing (Table 3). Less than 10% of respondents reported that HV had affected their personal health (Q21), led them to think about quitting their job (Q22), or made them feel unsafe in their work environment (Q25). Nearly a quarter (22.2%) of respondents reported that they could remember a specific time in the preceding 12 months when it had negatively impacted care for their patients (Q19), and 11.1% reported dreading coming to work due to being subjected to bullying (Q20). Furthermore, 65.6% of respondents indicated that they felt safe to report acts of HV in their hospital (Q23) and 32.2% of participants indicated it had previously been addressed by their institution (Q24).

### Horizontal Violence Prevalence in Subgroups: Gender, Position, and Experience

In the subgroup analysis, the behavior of being shouted at (Q4) was found to be more common among MLPs and females. However, as 77.8% of MLPs were female, making the variables of gender and position highly related, it cannot be determined whether this behavior is more prevalent among MLPs or females. Other behaviors that were more common in the female/MLP subgroups were being subjected to demeaning remarks (Q5), being a victim of unflattering rumors (Q11), feeling that coworkers did not respect their professional decisions (Q13), and being isolated or excluded by coworkers (Q14).

Several HV behaviors – such as being turned down or intentionally ignored when asking the opinion of a fellow coworker (Q10), being asked or hinted at to quit their job (Q15), set up to fail a task asked of them (Q16), and threatened for voicing their opinion (Q17) – were more common among attending physicians as well as those who were more experienced (number of years working). However, attending physicians also had the most experience working in the ED. Thus, it is not possible to determine whether these behaviors were more prevalent in the subgroup of position (attending physicians) or experience (number of years working).

## DISCUSSION

To the best of our knowledge, this study is the first to look at the prevalence of HV in the context of attending physicians, residents, and physician assistants in the ED. Previous research has largely focused on HV among nurses, but it was not clear whether these types of behaviors also extend into other healthcare professions. Prevalence data in this study ranged from 1.1% to 34.1%, which is similar to data previously published in nursing studies of 5%–38%.^2^,^3^,^8^,^9^,^12^ Nearly a quarter (22.2%) of participants felt that HV behaviors, either witnessed or experienced themselves, had negatively impacted patient care and 8.7% indicated that it had impacted their own health. Common behaviors (Q1, 2, 4, 5, 7, 11, and 12) identified in this survey fall into the previously described categories by Griffin of “Covert or overt verbal affront,” “Failure to respect the privacy of others,” and “Undermining clinical activities.”^11^ The prevalence of these behaviors having occurred “a few times, monthly, weekly, or daily” in the preceding 12 months ranged between 25 responses (27.4%) for Q12 (turned down when asking a co-worker to do a task) to 31 responses (34.1%) for Q4 (been shouted at). Only one respondent (1.1%) indicated having experienced or witnessed physical violence (Q18) between co-workers a few times in the preceding 12 months.

Previous studies have shown that HV behaviors impact patient care, medical errors, preventable adverse outcomes, negatively impact patient satisfaction, and increased malpractice risk.^1^ These results suggest that there is a potential opportunity to enhance patient care by improving the working environment for healthcare providers. Furthermore, the prevalence of these behaviors may even be higher than detected in this survey as only 65.6% of participants felt safe to report acts of HV to their institution, suggesting that employees may have refrained from participating in this study.

In an effort to identify successful interventions, a recent study from 2013 compared previously published reports of policy implementations addressing lateral violence from 12 sources. The authors concluded that the most important interventions include 1) an engaged and strong managerial leadership encouraging a supportive culture for policy changes addressing lateral violence; 2) involving administration and personnel “frequently and consistently including matters of lateral violence;” 3) “intentionally changing policy and environment;” and 4) “implementing multiple interventions simultaneously that may not be effective when used alone.”^15^ While more research is required to identify best practices, as much of the evidence comes from expert opinion, we believe the proposed implementations are financially feasible, can be implemented in a timely fashion, and will address both job satisfaction and the quality of patient care. Many institutions may already be in the process of addressing HV or LV among their nurse employees and may want to consider expanding their efforts to include physicians and physician assistants.

## LIMITATIONS

Our study has several limitations. The survey tool was not validated for a physician or MLP population. However, this tool was designed based on several survey tools that have been validated in studies examining the prevalence of HV in nurse and nursing student populations. We initially included Question 3 (“asked to do tasks below your competencies”) in this study as it had been used in previous studies, but we decided not to include responses to this question in the results or discussion part of this study as feedback pointed out that it may not fit the definition of HV depending on its interpretation. For transparency it was not removed from the figures and tables of this study.

This is a pilot study, and as such there were relatively small numbers of participants in each category. The study participants were from a single practice plan and a single residency. The authors intend to expand this study to include multiple institutions and residencies. The data may be influenced by a recall bias such that participants may not have accurately remembered all events in the preceding 12 months and some may have avoided filling out the survey if they did not feel safe in reporting HV behaviors. We therefore encourage future studies to limit participant recall of events to six months or less and stress the anonymity of responses. There may also have been selection bias. Those who participated may have done so because they have been victims of HV and wanted to report it, or conversely, perpetrators. Lastly, approximately 25% of potential participants did not complete the survey, resulting in a small sample size that may have skewed results.

## CONCLUSION

Horizontal violence and its impact on staff and patients is prevalent among emergency medicine attending physicians, residents, and MLPs. While direct comparisons to previously published data cannot be made due to the lack of a standardized survey tool, preliminary data suggest these behaviors extend beyond nursing to involve multiple healthcare professions. Everyday decisions made by physicians and physician assistants carry significant responsibility and may have a critical impact on the quality of care, medical errors, and outcomes of patients. Behaviors that negatively impact decision-making capacity should therefore be elicited and reduced or eliminated. Further research is warranted to understand and effectively intervene in behaviors that impact job satisfaction and patient care beyond the scope of nursing.

## Figures and Tables

**Figure:**
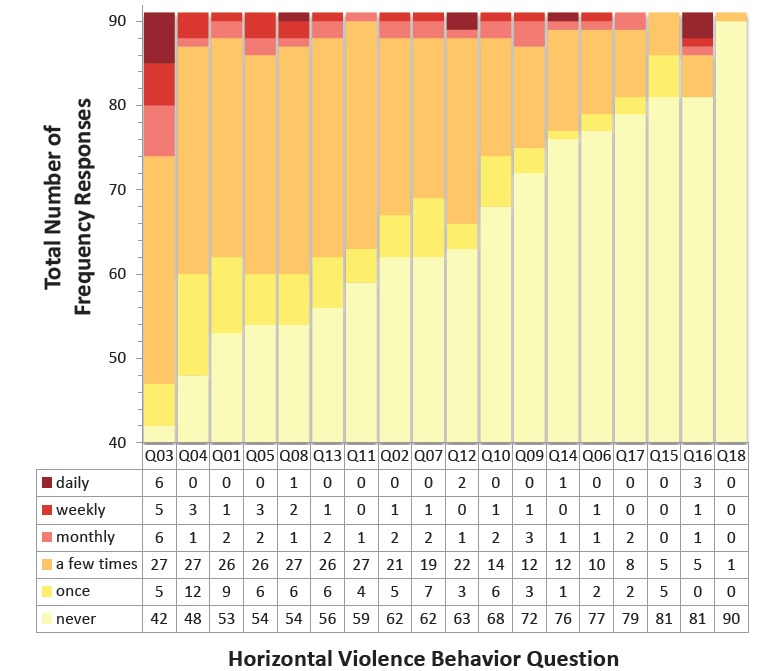
Number of frequency responses correlating to questions of Table 2. *Q,* question.

**Table 1:** Demographic data of participants (n=91) in a horizontal violence survey regarding the prevalence of workplace bullying, including physical assault, between emergency physicians, residents and mid-level providers.

Participant demographics	Results (n=91)
Gender	
Male	64.8%
Female	35.2%
Age (years)	
Under 21	1.1%
21–30	35.2%
31–40	39.6%
41–50	11%
51–60	8.8%
Over 60	4.4%
Position	
Attending physicians	41.8%
Residents	37.4%
MLPs	19.8%
Other (fellow)	1.1%
Experience (years)	
2 or less	45.1%
3–5	17.6%
6–10	20.9%
11–15	5.5%
Over 15	11%

*MLPs,* mid-level providers.

**Table 2:** Prevalence questions of survey used in this study of horizontal violence

Question (Q) #	Question content
Q1	Humiliated by a co-worker
Q2	Ridiculed by a co-worker for asking a question
Q3	Asked to do tasks below your competencies
Q4	Shouted at
Q5	Subject to demeaning remarks
Q6	Victim to threatening body language
Q7	Consistently criticized for your work
Q8	Deemed incompetent for a task within your skill level
Q9	Felt pressured to change your professional opinion or treatment plan due to feeling intimidated by another co-worker
Q10	Turned down or intentionally ignored when asking the opinion of a fellow co-worker
Q11	Victim of unflattering rumors
Q12	Turned down when asking a co-worker to do a task
Q13	Feel that your co-workers do not respect your professional decisions
Q14	Isolated or excluded by co-workers
Q15	Asked or hinted at to quit your job
Q16	Set up to fail a task asked of you (such as completing a task in a time frame that is not possible or realistic)
Q17	Threatened for voicing your opinion
Q18	Physically assaulted

*Q,* question.

**Table 3:** Responses to questions 19–25 eliciting impact of horizontal violence.

Question (Q)#	Question content
Q19	Can you remember a specific time at which acts of horizontal violence have affected care for your patients?
Q20	Did you or do you ever dread coming to work due to being subjected to bullying at the workplace?
Q21	Has Horizontal Violence (verbal or non-verbal) affected your own health?
Q22	Have you ever or are you currently thinking about quitting your job due to acts of Horizontal Violence towards you?
Q23	Do you feel safe to report acts of Horizontal Violence in your hospital?
Q24	Has your current institution addressed horizontal violence in the past year?
Q25	Do you feel unsafe in your current work environment for any reason?

*Q,* question.
